# Cross-cultural adaptation and validation of the Spanish version of the Prevent for Work questionnaire

**DOI:** 10.3389/fpubh.2024.1453492

**Published:** 2025-01-07

**Authors:** Julia Blasco-Abadía, Pablo Bellosta-López, Víctor Doménech-García, Thorvaldur Skuli Palsson, Steffan Wittrup McPhee Christensen, Morten Hoegh, Pedro Berjano, Francesco Langella

**Affiliations:** ^1^Facultad de Ciencias de la Salud, Universidad San Jorge, Zaragoza, Spain; ^2^Department of Physiotherapy and Occupational Therapy, Aalborg University Hospital, Aalborg, Denmark; ^3^Department of Health Science and Technology, Aalborg University, Aalborg, Denmark; ^4^Department of Physiotherapy, University College of Northern Denmark, Aalborg, Denmark; ^5^Department of GSpine4, IRCCS Ospedale Galeazzi-Sant’Ambrogio, Milan, Italy

**Keywords:** musculoskeletal disorders, disabling pain, occupational setting, forward-backward translation, face validity, known-groups validity, reliability, minimal detectable change

## Abstract

**Background:**

Musculoskeletal pain represents an increase in medical expenses due to disability and decreased quality of life among workers. Various biopsychosocial factors contribute to the development of persistent and disabling musculoskeletal pain. The Prevent for Work questionnaire (P4Wq) intended to analyze these factors. In this study, the original Italian version of the P4Wq was translated and culturally adapted to Spanish. Moreover, the psychometric properties were evaluated among Spanish workers with and without recent history of disabling spinal pain.

**Methods:**

The first phase consisted of a forward-and-backward translation process and evaluating the face-validity of the questionnaire among 30 Spanish workers. The second phase involved 153 Spanish workers who completed the P4Wq, Oswestry Disability Index (ODI), and EQ-5D-5L questionnaires. Finally, 50 Spanish workers completed the P4Wq 2 weeks later to evaluate test–retest reliability and measurement error.

**Results:**

Minor changes were made after the forward-and-backward translation process, which ensured that the Spanish versions was face-valid. The P4Wq demonstrated acceptable internal consistency for Spanish version (Cronbach’s alpha: 0.91), a moderate negative association with the indicator of quality of life (*ρ* < −0.39; *p* = 0.001) and moderate positive association with the disability index (*ρ* > 0.46; *p* = 0.001). Furthermore, the P4Wq showed good to excellent item response stability (weighted kappa = 0.75–0.96) and good for the total score (ICC = 0.98).

**Conclusion:**

The Spanish version of the P4Wq was face-valid and exhibited a similar structure as the original version. Additionally, good internal consistency and construct validity were found. This translated version of the questionnaire can therefore be considered acceptable for use by workers with and without history of disabling musculoskeletal pain.

## 1 Introduction

Musculoskeletal (MSK) disorders are the leading causes of disability and functional limitations in daily life and work participation worldwide (1). The impact on public health is considerable (2), requiring new ways of managing these conditions (3).

Pain is the cardinal symptom of MSK disorders and represents a challenge in occupational settings, serving as primary reason for increased medical expenditures tied to disability and compromised quality of life of workers (4). In a European context, MSK pain is responsible for more than half of work-absenteeism (5). This negative effect demonstrates that the repercussions of MSK pain extend beyond individual well-being, bearing significant economic consequences for employees, employers, and society at large (5, 6).

Multiple factors such as older age, unhealthy lifestyle, compromised mental health, comorbidities, and the presence of MSK symptoms contribute to the development and persistence of MSK pain, limiting daily activities and work capacity (3). There are also risk factors for MSK pain specific to the occupational setting (7), including physical work demands such as manual handling or awkward postures, as well as organizational factors related to an unhelpful workplace design, and poor job satisfaction (8). The interconnection of these factors underscores the multifaceted nature of work-related MSK pain and emphasizes the importance of a broad assessment of this condition.

Identifying modifiable risk factors is key to estimate the likely trajectory of a health condition, thus aiding healthcare professionals in making informed management decisions (9, 10). Moreover, an early identification of potential risk factors can aid in preventing the onset and persistence of chronic MSK pain (10, 11). While previous research has predominantly explored the correlation between self-reported work ability and return to work in rehabilitation settings, there is a urgent need for tools that comprehensively assess biopsychosocial factors associated with MSK pain in the occupational settings (12). Accurate and transparent information regarding workers’ conditions is essential for devising effective rehabilitation strategies (13, 14) and facilitating a successful return to work (15–17). The Prevent for Work questionnaire (P4Wq) builds on MSK pain within a biopsychosocial framework (18).

The P4Wq was developed under a European initiative between Italian, Spanish and Danish institutions aiming to develop a self-administered questionnaire that assesses factors related to the development of work-related MSK pain (18). While its adoption can facilitate a comprehensive understanding of the multifaceted nature of work-related MSK pain and aid in devising effective prevention and management, objective data on its adequate psychometric properties only exist in an Italian working population (19). The original Italian version provided a concise measure of risk factors for work-related back disorders that have demonstrated good content validity, construct validity, internal consistency reliability and high face validity. To expand this to other languages, it is important to translate and culturally adapt the questionnaire to other languages. In the future, this will allow for a comparison of work-related MSK pain in other populations of workers from different European countries.

This study aimed to cross-culturally adapt the original Italian version of the P4Wq into European-Spanish, as well as evaluate its psychometric properties (i.e., face validity, structural validity, internal consistency, construct validity, floor and ceiling effects, reliability, and measurement error) in workers.

## 2 Materials and methods

### 2.1 Study design

This study was set up in two phases: (i) a cross-cultural adaptation phase and (ii) a validation phase. The present study was conducted in consensus with the COnsensus-based Standards for the selection of health status Measurement INstruments (COSMIN) study design checklist (20). The study was conducted per the Helsinki Declaration and the study protocol was approved by the ethics committee at San Jorge University (N01-20/21). All participants provided informed consent before taking part in the study.

### 2.2 Study settings and participants

Participants were among active workers recruited in February–May 2021 from institutions in the autonomous community of Aragon (Spain), as part of the Prevent4Work project (4). Inclusion criteria established were (a) age between 18 and 65 years old, (b) ability to read and speak Spanish, and (c) one or more year of employment in the current position. People with (a) previous history of major surgery for a MSK disorder, (b) diagnosis of any persistent painful condition of specific pathology (e.g., rheumatoid arthritis, fibromyalgia, spinal stenosis), (c) habitual intake of antidepressants, and (d) medial history involving cancer, brain or spinal cord injury, or psychiatric and neurological disorders, were excluded. Notwithstanding the prior briefing about the criteria, each participant completed a self-reported checklist at the initiation of the questionnaire series to verify their compliance with the outlined selection criteria.

### 2.3 Cross-cultural adaptation

The translation and cross-cultural adaption was done following forward-and back-translation (21) from Italian into European-Spanish. This process involved two proficient translators, one with a medical background, who were native speakers of European-Spanish. They independently generated two translations of the original questionnaire from Italian to European-Spanish. Subsequently, these translations were compared and analyzed to detect any inconsistencies between them. These inconsistencies in the translations were discussed until a consensus on the final version was reached. In case of disagreements, a member of the research group (PB-L) was consulted to reach an agreement. Following this, a backward translation of the synthesized version was carried out from Spanish back to Italian by two independent native Italian translators, who were not previously familiar with the original Italian version of the questionnaire. Subsequently, an expert committee composed of the authors, convened to assess the final version of the translated questionnaire. The committee reviewed the entire forward-and back-translation process, specifically examining any potential inconsistencies and ensuring that the questions were comprehensible across the target populations.

### 2.4 Pilot study for face-validity

After the creation of the prefinal versions of the P4Wq, a group of thirty volunteer workers participated in pilot testing to assess its face validity. Each participant completed the questionnaire and subsequently underwent an interview, where the focus was on examining their understanding of each questionnaire item and the available responses. To quantify the face validity, two distinct 5-point Likert scales were employed to evaluate the clarity and comprehensibility of the questionnaire items. The face validity index was determined as the average value of the Likert scales, converted to a scale ranging from 0 (total lack of clarity or comprehension) to 100 (complete clarity or comprehension) (22); with a score > 80% considered satisfactory (23). Furthermore, completion time was registered.

### 2.5 Validity study

Participants completed a paper version of the Spanish version of the P4Wq, a questionnaire designed to assesses biopsychosocial factors related to the development of work-related MSK pain. The P4Wq includes sociodemographic data, disabling pain prevalence, and potential risk factors such as job satisfaction, mental stress, kinesiophobia, catastrophizing, and physical stress, with responses scored on a 5-point Likert scale where higher scores indicate greater risk. The Spanish version of the EuroQol Five-Dimensions Five-Levels levels (EQ-5D-5L) (24, 25) was used to evaluate general health status across five dimensions (mobility, self-care, usual activity, pain-discomfort, and anxiety-depression) using a 5-point scale, alongside a visual analogue scale (EQ-VAS) for overall health where higher scores represent better health. Lastly, the Oswestry Disability Index (ODI) (26, 27) was used to measure disability related to spinal disorders through 10 dimensions of daily activities, scored on a 6-point scale, with higher scores reflecting greater levels of disability. A detailed description of the questionnaires is provided in Supplementary Table A.

A subset of 50 individuals was randomly chosen to undergo a test–retest assessment of the Spanish version of the P4Wq after a 2-week interval, aimed at evaluating the test–retest reliability and measurement error. During this stage, all participants were asked to fill out a checklist to ensure the consistency of their responses over the interim period. This checklist included questions regarding the occurrence of any new episodes of disabling MSK pain, instances of sick leave, or the reception of physical or psychological treatment.

### 2.6 Hypotheses testing for construct validity

As contemplated in the COSMIN recommendations, construct validity was assessed by means of convergent validity and known-groups validity (20). Following a similar methodology to the original development of the P4Wq to evaluate convergent validity, we hypothesized that a significant moderate correlation would exist between the total scores of the Spanish version of the P4Wq, and existing measures of quality of life (i.e., the visual analogue scale of the EQ-5D-5L) and disability (i.e., the ODI). Furthermore, as the P4Wq was intended to measure risk factors for work-related MSK disorders in the spine (neck, thoracic, and low back regions), data from participants reporting 12 months prevalence of disabling spinal MSK pain were extracted for further analysis (i.e., disabling spinal pain vs. no disabling spinal pain). Disabling pain refers to pain that has limited daily activities (28). We hypothesized that workers with a history of disabling spinal pain would present higher scores in the P4Work compared to those workers classified as having no disabling spinal pain.

### 2.7 Sample size

The COSMIN recommendations determined the sample size for calculating the confirmatory factor analysis, which is considered an excellent sample size when it consists of 7 times the number of items (i.e., 140 participants) (20). However, after accounting for up to 10% of ineligible questionnaires, a sample size of 154 participants was intended. In fact, a sample size higher or equal to 100 participants was considered sufficient for testing internal consistency, construct validity or test–retest reliability, while was considered adequate if higher or equal to 50 participants (20).

### 2.8 Statistical analysis

Statistical analyses were performed using SPSS v.28 (IBM, Chicago, IL, USA), except for the confirmatory factor analysis, using STATA v.18.0 (StataCorp, College Station, TX, USA). Participant data containing blank items in the P4Wq were excluded from the analysis. Mardia’s test was used to determine whether P4Wq data were multivariate normally distributed. The results were expressed as mean (± standard deviation (SD)), and/or 95% confidence intervals (CI). The level of significance was set at *p*-value <0.05. The detailed description of the statistical tests used in the present study is depicted in Table 1, for: (1) structural validity (29–33); (2) internal consistency (34); (3) construct validity by means of convergent validity (35), and known groups validity (36); (4) test–retest reliability (37, 38); (5) measurement error (39); and (6) floor and ceiling effects (40). Subgroup analyses were conducted for sex, age category (<45 years or ≥ 45 years) and work type (office, healthcare, blue-collar) using linear regression models and independent T-Student or ANOVA with Bonferroni correction as *post hoc* test.

**Table 1:** Statistical tests used.

Psychometric property	Data	Statistical test	Purpose	Criteria for interpretation
Structural validity	P4Wq 20 items	Bartlett’s test of sphericity	To assess the adequate composition of the sample	Adequate if *p* < 0.05
		Kaiser-Meyer-Olkin		Adequate if KMO > 0.7
		Confirmatory factor analysis^#^	To assess the dimensionality of the questionnaire	Items with factor loadings >0.4 were considered acceptable.
		Goodness of fit statistics: SRMR. RMSEA. CFI. TLI.	To evaluate how well the proposed model fits the observed data and to assess the adequacy of the chosen dimensional structure	SRMR: <0.08 “good fit,” >0.08 “poor fit.” RMSEA: <0.08 “good fit,” 0.08 to 0.1 “adequate fit,” >0.1 “poor fit.” CFI&TLI: >0.9 “good fit,” 0.9 to 0.8 “adequate fit,” <0.8 “poor fit.”
Internal consistency	P4Wq 20 items	Cronbach’s α	To assess the accuracy and consistency of the questionnaire	Acceptable if *α* > 0.7
		Cronbach’s α if item deleted		Analyze variations from to the original *α* value
		Corrected item-total correlation	To assess the correlation between the item score and the scale score minus the contribution of that item to the score	Acceptable if *r* > 0.3
Construct validity – Convergent validity	P4Wq total score, EQ-VAS, ODI	Pearson’s correlation	To evaluate the correlation between P4Wq total score with EQ-VAS & ODI	Pearson’s coefficient *p*: >0.70 “strong,” 0.40 to 0.69 “moderate,” 0.10 to 0.39 “weak,” <0.10 “negligible.”
Construct validity – Known-groups validity	P4Wq total score and subdomains	Student T-Tests	To explore the differences in P4Wq total scores and subdomains sub-scores.	Differences between groups are present if *p* < 0.05.
		Cohen’s d	To assess the effects sizes of group differences	*d*: >0.8 “large effect,” 0.79 to 0.5 “moderate effect,” <0.49 “small effect”
Test–retest reliability	P4Wq total score and subdomains	Weighted Kappa correlation coefficient of agreement	To assess item response stability	*k*: 0.81 to 1.0 “excellent,” 0.61 to 0.80 “good,” 0.41 to 0.60 “moderate,” 0.21 to 0.40 “fair,” <0.20 “poor.”
		ICC with a 95% confidence interval^§^	To assess the reliability of the total score and subdomains sub-scores	ICC: >0.90 “excellent,” 0.90 to 0.75 “good,” 0.75 to 0.50 “moderate,” <0.50 “poor”
Measurement error	P4Wq total score and subdomains	SEM	To evaluate the variation of the sample mean	NA
		MDC_95_	To calculate the smallest change beyond the margin of error for research purposes	NA
		MDC_90_	To calculate the smallest change in ordinary clinical perspective	NA
Floor and ceiling effects	P4Wq total score	Frequency distribution of the total scores	To indicate whether the instrument is able to accurately measure the full range of the construct being assessed	Absence of floor and ceiling effects with a cut-off point of up to 15% within the highest or lowest scores

SRMR, Standardized Root Mean Square Residual; RMSEA, Root Mean Square Error of Approximation; CFI, Comparative Fit Index; TLI, Tucker-Lewis index; SEM, standard error of the mean; ICC, intraclass correlation coefficient; MDC, minimum detectable change.

^#Confirmatory factor analysis using the robust maximum likelihood estimation with Satorra–Bentler adjustments.^

^§Intraclass correlation coefficient with a 95% confidence interval following a two-way mixed effects model with absolute agreement.^

## 3 Results

### 3.1 Cross-cultural adaptation and face-validity processes

There were no considerable differences between the forward translations of the original Italian version of the P4Wq into European-Spanish, except for minor variations in word order that did not alter the meaning of the items. Similarly, there were no major discrepancies between the backward translations, preserving the meaning of the original version.

Thirty workers in Spain (40.7 ± 9.2 years, 50% females) completed the prefinal version of the P4Wq. The median time to fill the P4Wq was 4 min [IQR 3–5] for the Spanish version. The face validity index was 93.8%. No clarity and comprehension difficulties were reported for the Spanish with all items scored 4 or 5 (i.e., clear or very clear). No further changes were implemented, and the final version of the P4Wq in European-Spanish is presented in the Supplementary material.

### 3.2 Participants characteristics for the validity study

Table 2 presents the sociodemographic and questionnaire scores of 153 out of 154 workers in Spain who completed the Spanish version of the P4W. One worker, for unknown reasons, did not complete all the items and was removed from the analysis. A total of 119 (77%) workers reported disabling musculoskeletal pain in the spinal region (i.e., neck, thoracic, or low back) in the previous 12 months, with a median pain intensity of 4 out of 10 [IQR 3–5].

**Table 2:** Characteristics of the participants.

	
Age (years)	41.4 ± 11.6
Female *n* (%)	96 (63%)
Weight *(kg)*	68.7 ± 12.7
Height *(cm)*	168.5 ± 8.4
Body Mass Index (kg/m^2^)	24.1 ± 3.8
Current position (years)	9.4 ± 7.6
Work type *n* (%)	
Healthcare workers	56 (37%)
Office workers	42 (27%)
Blue-collar workers	55 (36%)
Disabling MSK pain in the last 12 months *n* (%)	
Neck	82 (54%)
Shoulders	66 (43%)
Elbow	21 (14%)
Wrist/Hand	44 (29%)
Dorsal region	41 (27%)
Low back	82 (54%)
Hip	30 (20%)
Knee	47 (31%)
Ankle/Foot	19 (12%)
Regions affected per worker	2 [1–4]
EQ-5D-5L	
General Health – VAS (0/100)	81.3 ± 16.2
Mobility (0/4)	0.13 ± 0.34
Self-care (0/4)	0.03 ± 0.22
Usual activity (0/4)	0.19 ± 0.42
Pain-Discomfort (0/4)	0.75 ± 0.70
Anxiety-Depression (0/4)	0.28 ± 0.53
Oswestry Disability Index	
Total score (0/100)	8.1 ± 7.9
P4Wq	
Total score (0/80)	26.8 ± 11.8
JSS (0/16)	4.7 ± 3.0
MSS (0/24)	7.8 ± 4.2
KCS (0/16)	5.8 ± 4.1
PSS (0/24)	8.6 ± 5.7

Data are expressed in median ± standard deviation unless otherwise specified; MSK, muskuloskeletal; P4Wq, Prevent4Work questionnaire; JSS, job satisfaction subdomain; MSS, mental stress subdomain; KCS, kinesiophobia & catastrophizing subdomain; PSS, physical stress subdomain; VAS, visual analogue scale of the EuroQol questionnaire for self-percieved health status.

### 3.3 Structural validity

Kaiser-Meyer-Olkin and Bartlett’s Test of Sphericity demonstrated adequate sample composition for the questionnaire (KMO = 0.872; *p* < 0.001) versions. The confirmatory factor analysis showed a four-factor model with almost all factor loadings greater than 0.40, confirming the biopsychosocial dimensions of the questionnaire with the 4 pre-established domains. The factor loadings of the different items are presented in Table 3.

**Table 3:** Confirmatory factor analysis, internal consistency indicators, and item’s reliability of the Spanish version of the P4Wq.

	Weighted Kappa (*n* = 50)	Corrected item–total correlation	Cronbach’s alpha if item deleted	Components			
Items label				JSS	MSS	KCS	PSS
1-Does your job give you the opportunity to improve your skills?	0.85	0.45	0.91	0.64			
2-Do you feel motivated by and involved in your work?	0.81	0.54	0.91	0.82			
3-Is there a satisfactory level of cooperation with your colleagues?	0.78	0.65	0.90	0.90			
4-Are you satisfied with the people you work with?	0.77	0.64	0.90	0.94			
5-In the last 4 weeks, have you felt calm and peaceful?	0.76	0.49	0.91		0.60		
6-I find it difficult to relax and enjoy myself.	0.75	0.50	0.91		0.66		
7-I find it difficult to deal with other people.	0.90	0.56	0.90		0.63		
8-I find it difficult to feel happy.	0.84	0.51	0.91		0.69		
9-In the last 4 weeks, have you had problems concentrating?	0.75	0.47	0.91		0.77		
10-In the last 4 weeks, have you found it difficult to think clearly?	0.82	0.54	0.91		0.81		
11-I avoid unnecessary movements to prevent the pain from getting worse.	0.79	0.49	0.91			0.70	
12-I fear the pain might increase.	0.84	0.64	0.90			0.81	
13-I feel as if I could no longer bear the pain.	0.81	0.68	0.90			0.94	
14-The pain is terrible, and I think it will never get better.	0.83	0.66	0.90			0.90	
15-Does your job involve having to lift heavy weights (over 5 kg)?	0.96	0.39	0.91				0.63
16-Does your job involve having to lift loads from an uncomfortable position?	0.96	0.65	0.90				0.84
17-Does your job involve having to simultaneously bend and rotate your trunk?	0.82	0.58	0.90				0.76
18-Does your job involve having to lift your arms above the shoulder height?	0.91	0.43	0.91				0.65
19-Do you have to keep uncomfortable postures at work?	0.90	0.66	0.90				0.83
20-Do uncomfortable postures at work prevent you from applying enough strength?	0.88	0.65	0.90				0.81

*N = 153. P4Wq, Prevent4Work questionnaire; JSS, job satisfaction subdomain; MSS, mental stress subdomain; KCS, kinesiophobia & catastrophizing subdomain; PSS, physical stress subdomain. Weighted kappa values represent items’ reliability after a 2-week period. Component values represent items’ weights after the robust maximum likelihood estimation with Satorra-Bentler adjustments.*

All the goodness-of-fit statistics indicated a ‘good fit’ for the Spanish version of the P4Wq (Standardized Root Mean Square Residual, SRMR = 0.074; Root Mean Square Error of Approximation, RMSEA = 0.07; Comparative Fit Index, CFI = 0.93; Tucker–Lewis Index, TLI = 0.92).

### 3.4 Internal consistency

The internal consistency for the total score of the P4Wq was acceptable according to the Cronbach’s alpha values (*α* = 0.91). Similarly, acceptable internal consistency was found for the domains sub-scores (α_JSS_ = 0.90; α_MSS_ = 0.85; α_KCS_ = 0.91; α_PSS_ = 0.88). Corrected item-total correlations and Cronbach’s α if item deleted was calculated are presented in Table 3.

### 3.5 Convergent validity

**Figure 1:**
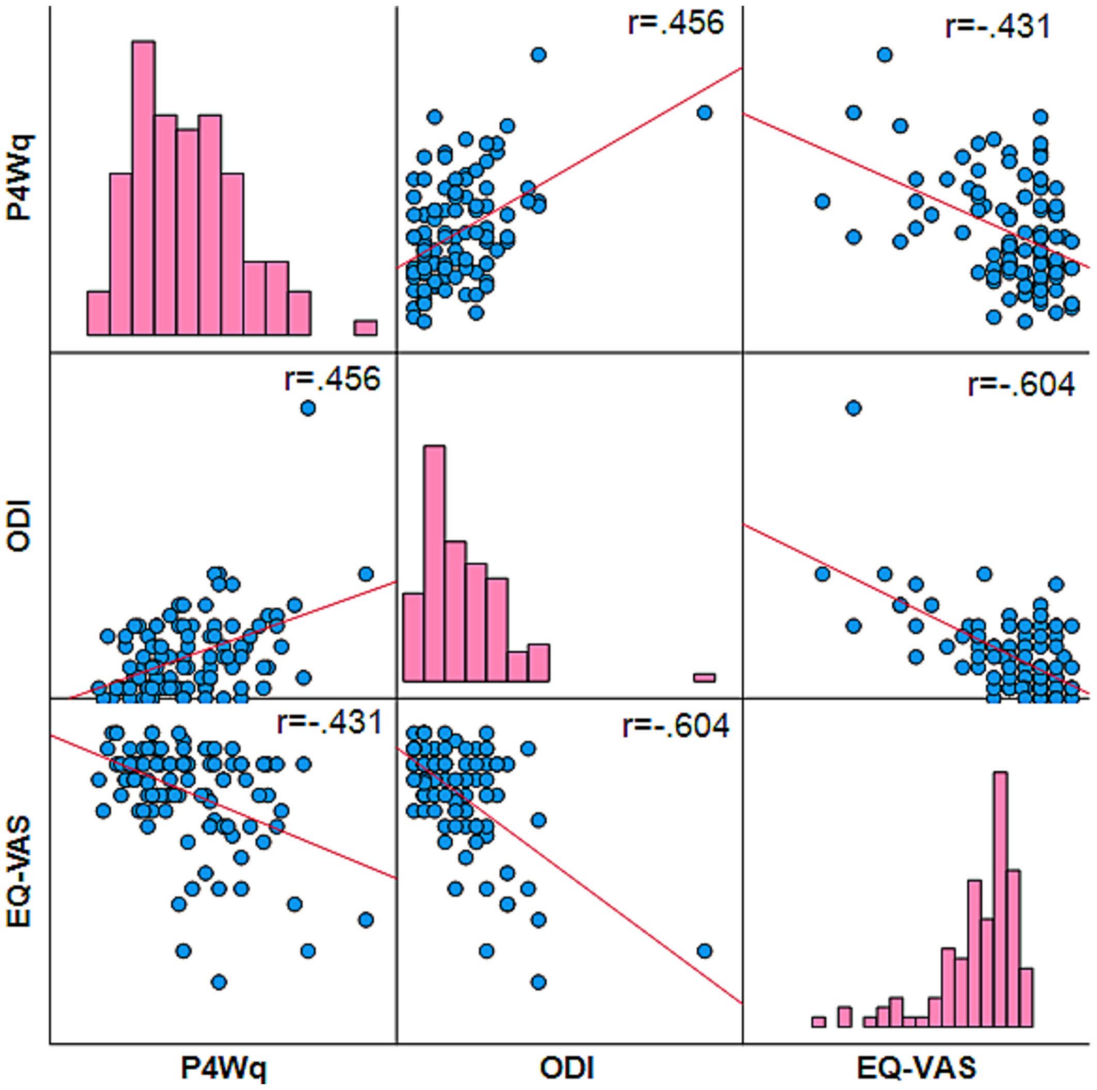
Scatter plots and histograms for the P4Wq, ODI, and EQ-VAS. P4Wq, Prevent for Work Questionnaire; ODI, the Oswestry Disability Index; EQ-VAS, visual analogue scale for the general health status of the EuroQol questionnaire. The associations were statistically significant for all variables after Pearson’s correlation test (two-tailed, *p* ≤ 0.001).

The P4Wq total score showed a moderate negative association with the indicator of quality of life (i.e., EQ-VAS) (*ρ* = −0.43; *p* < 0.001) and moderate positive association with the disability index (i.e., ODI) (*ρ* = 0.46; *p* < 0.001). Figure 1 represents the score distribution and between variables association.

### 3.6 Known-groups validity

For the Spanish workers, the “disabling spinal pain” group showed higher total score (mean difference = 12.1; *p* < 0.001; *d* = 1.10) and the subscales of job satisfaction (mean difference_JSS_ = 3.0; *p* < 0.001; *d* = 0.80), mental stress (mean difference_MSS_ = 3.7; *p* < 0.001; *d* = 0.90), kinesiophobia & catastrophizing (mean difference_KCS_ = 2.8; *p* = 0.001; *d* = 0.70), and physical stress (mean difference_PSS_ = 2.5; *p* = 0.022; *d* = 0.50) domains compared with the “no disabling spinal pain” group. Descriptive statistics depicted by the group are presented in Table 4.

**Table 4:** P4Wq scores comparisons between participants with and without history of disabling spinal pain in the last 12 months.

	Disabling spinal pain (*n* = 119)	No disabling spinal pain (*n* = 34)	*p* value
Total score (0/80)	33.3 ± 14.2	21.2 ± 8.9	<0.001
JSS (0/16)	6.7 ± 4.2	3.6 ± 2.9	<0.001
MSS (0/24)	9.9 ± 4.8	6.2 ± 3.5	<0.001
KCS (0/16)	6.5 ± 4.3	3.7 ± 4.1	0.001
PSS (0/24)	10.2 ± 5.8	7.6 ± 5.0	0.022

Data are expressed in median ± standard deviation. P4Wq, Prevent4Work questionnaire; JSS, job satisfaction subdomain; MSS, mental stress subdomain; KCS, kinesiophobia & catastrophizing subdomain; PSS, physical stress subdomain. The “disabling spinal pain” group consisted of workers reporting disabling musculoskeletal pain in the last 12 months in the spinal region (i.e., neck, dorsal, and/or low back region). *p* values represent significance level after independent samples T-test.

### 3.7 Floor and ceiling effects

The analyses of the distribution of frequencies regarding the total score indicated an absence of floor or ceiling effects, presented in Figure 1. No responders achieved the highest or lowest scores in the total score.

### 3.8 Test–retest reliability and error of measurement

Test–retest reliability and error of measurement were tested. More specifically, fifty workers (36.8 ± 10.1, 64% females) completed the test–retest after a two-week period (14 ± 2 days).

The test–retest reliability was good to excellent for all items with the weighted Kappa coefficients ranging from 0.75 to 0.96 as presented in Table 3.

The test–retest reliability was ‘good’ for the total score (ICC = 0.98) and JSS (ICC = 0.91), MSS (ICC = 0.94), KCS (ICC = 0.96), and PSS (ICC = 0.98) domains. The specific values of the ICC (3,1) with 95% confidence interval, SEM, MDC_95_ and MDC_90_ for the total scores and domains sub-scores of the P4Wq are presented in Table 5.

**Table 5:** Test–retest reliability and measurement error indicators.

	ICC _(3,1)_ (95% CI)	SEM		MDC_95_		MDC_90_	
		raw score	Cohen’s *d*	raw score	Cohen’s *d*	raw score	Cohen’s *d*
Total score (0/80)	0.98 (0.97–0.99)	1.7	0.14	4.6	0.39	3.9	0.33
JSS (0/16)	0.91 (0.84–0.95)	0.9	0.30	2.5	0.83	2.1	0.70
MSS (0/24)	0.94 (0.90–0.97)	1.0	0.23	2.9	0.69	2.4	0.57
KCS (0/16)	0.96 (0.94–0.98)	0.8	0.19	2.3	0.56	1.9	0.46
PSS (0/24)	0.98 (0.96–0.99)	0.8	0.14	2.2	0.39	1.9	0.33

ICC_(3,1)_: intraclass correlation coefficient for a two-way mixed effects model with absolute agreement; CI, confidence interval; SEM, standard error of measurement; MDC_95_, minimum detectable change at 95% of confidence; MDC_90_, minimum detectable change at 90% of confidence; JSS, job satisfaction subdomain; MSS, mental stress subdomain; KCS, kinesiophobia & catastrophizing subdomain; PSS, physical stress subdomain.

### 3.9 Subgroups analysis

Subgroups analysis for sex, age categories, and work type are detailed in the Supplementary material. All subgroups showed comparable levels of adequate internal consistency and convergent validity.

The linear regression models showed that the work type was associated with the P4Work total score (*p* = 0.003), and the domains of KCS (*p* = 0.004) and PSS (*p* < 0.001). The linear regression models showed no associations with sex or age categories. Specifically, office workers showed lower total P4Work score and PSS domain than healthcare workers and blue-collar workers (*p* < 0.001), while blue-collar workers showed higher scores in the KCS domain than office workers (*p* = 0.010).

## 4 Discussion

The objective of this study was to translate and culturally adapt the original Italian version of the P4Wq into European-Spanish, and subsequently assess its psychometric properties in a working population. This version demonstrated good face validity, structural validity, internal consistency, construct validity, known-groups validity, floor and ceiling effects, and test–retest reliability.

### 4.1 Cross-cultural adaptation and face-validity processes

The translation of the P4Wq underwent a rigorous and systematic process to ensure semantic equivalence with the original version in Italian. Spanish participants demonstrated adequate interpretation and understanding of all questionnaire items, with no items requiring special attention. Minor discrepancies encountered in the forward-and backward-translation process, primarily related to the use of verbs and synonymous words, are considered normal in translations and cultural adaptations of questionnaires (41) and were resolved by the group.

### 4.2 Structural validity and internal consistency

The four-factor solution for the Spanish version closely resembled the results obtained in the original P4Wq version, in which the four subdomains (JSS, MSS, KCS, and PSS) comprehensively assess the worker from a biopsychosocial perspective (18, 19). Additionally, all goodness-of-fit statistics indicated a ‘good fit’ for the Spanish version.

Moreover, the internal consistency values, reflected by the Cronbach’s *α*, resembled the original version for the total score (*α* = 0.89) and domains sub-scores (0.82 < *α* < 0.91) (19).

### 4.3 Construct validity and floor/ceiling effects

In line with the original version with Italian workers, the total scores of the Spanish version of P4Workq showed a moderate positive correlation with the ODI and a moderate negative correlation with the EQ-VAS. Based on the analysis in the sample of Spanish workers, the P4Wq appears to mitigate the floor effect observed in the ODI and the ceiling effect seen in the EQ-VAS (19). This result suggests a higher sensitivity of the P4Wq for classifying populations of active workers. Furthermore, the P4Wq offers promising ability to stratify differentiate between healthy workers with a history of disabling spinal pain, which could be predictive of future musculoskeletal pain events or long-term sick leave from work (42). Additionally, the differences in effect sizes between groups would be considered as moderate-large for the total score and most domain sub-scores, which were at or above the SEM. However, despite being statistically significant, the physical stress subdomain showed a lower discriminative ability between groups compared to the rest of the subdomains. These differences not only in the physical domain reinforces the biopsychosocial view of MSK pain, which extends beyond exclusively physical and ergonomic factors (18), which reinforces the P4Wq ability to capture the multifactorial nature of work-related pain.

### 4.4 Test–retest reliability and error of measurement

For the reliability results after 2 weeks, item response stability demonstrated good to excellent reliability for all items based on the weighted Kappa coefficients (43), comparable to the values obtained in the original version (19).

Notably, this study is the first to evaluate test–retest reliability for the P4Wq, finding a moderate to excellent reliability for both the total and domain sub-scores, and allowing the calculation of the SEM and the MDC to be considered in future studies. In this study, both the MDC_90_ and MDC_95_, ranged between 4 to 5 points for the total score and between 2 to 3 points for the domain subs-cores, suggesting that the smallest detectable change would lie between 6 to 13% of the maximum scores.

### 4.5 Clinical implications

The validation of the P4Wq into Spanish extends its utility beyond the original Italian population, offering a reliable and culturally adapted tool for Spanish workers. Given the multifaceted nature of work-related MSK pain, this questionnaire allows healthcare professionals to assess key factors contributing to MSK pain within a biopsychosocial framework. For example, the questionnaire allows for early identification of workers at risk of developing pain indifferent activity sectors (e.g., office workers, healthcare workers, blue-collar workers). Moreover, the Spanish version of the P4Wq supports cross-cultural research initiatives and the development of evidence-based occupational policies to reduce MSK pain and improve workers’ health.

### 4.6 Limitations

The primary limitation of the Spanish version of the P4Wq was the exclusion of workers on sick leave from the study sample, restricting the generalizability of results to the active working population. Given the association between negative pain beliefs and early withdrawal from the labor market (44), future studies should assess the psychometric properties of the Spanish version of the P4Wq in samples of workers on sick leave, and also in workers with self-reported chronic spinal pain. More importantly, a broad implementation of the questionnaire in working population would be valuable as this might help understanding whether the questionnaire can identify workers at risk of developing debilitating MSK pain. Future evaluations should thus include responsiveness and predictive capacity for new occurrences of MSK pain or long-term sick leave (42).

## 5 Conclusion

In conclusion, the Spanish version of the P4Wq was face-valid and exhibited a similar structure to the original version, as well as good internal consistency and construct validity. Furthermore, the Spanish version exhibited excellent test–retest reliability and, for the first time, provided values for measurement error. Additionally, it is suitable for use within the active working population and are valuable tools for a brief yet comprehensive biopsychosocial evaluation of factors related to the development of work-related MSK pain.

## Data Availability

The original contributions presented in the study are included in the article/Supplementary material, further inquiries can be directed to the corresponding author.

## References

[1] Krishnan KS Raju G Shawkataly O. Prevalence of work-related musculoskeletal disorders: psychological and physical risk factors. Int J Environ Res Public Health. (2021) 18:9361. doi: 10.3390/ijerph18179361, PMID: 34501950 PMC8430476

[2] Briggs AM Woolf AD Dreinhöfer K Homb N Hoy DG Kopansky-Giles D. Reducing the global burden of musculoskeletal conditions. Bull World Health Organ. (2018) 96:366–8. doi: 10.2471/blt.17.204891, PMID: 29875522 PMC5985424

[3] Tousignant-Laflamme Y Houle C Longtin C Gérard T Lagueux E Perreault K. Prognostic factors specific to work-related musculoskeletal disorders: an overview of recent systematic reviews. Musculoskelet Sci Pract. (2023) 66:102825. doi: 10.1016/j.msksp.2023.102825, PMID: 37463542

[4] Bellosta-López P Domenech-Garcia V Palsson TS Christensen SW Silva PB Langella F. European knowledge alliance for innovative measures in prevention of work-related musculoskeletal pain disorders (prevent 4Work project): protocol for an international mixed-methods longitudinal study. BMJ Open. (2021) 11:e052602. doi: 10.1136/bmjopen-2021-052602, PMID: 34521678 PMC8442076

[5] de Kok J Vroonhof P Snijders J Roullis G Clarke M Peereboom K. Work-related musculoskeletal disorders: Prevalence, costs and demographics in the EU. Luxembourg: European Agency for Safety and Health at Work (2019).

[6] Keyaerts S Godderis L Delvaux E Daenen L. The association between work-related physical and psychosocial factors and musculoskeletal disorders in healthcare workers: moderating role of fear of movement. J Occup Health. (2022) 64:e12314. doi: 10.1002/1348-9585.12314, PMID: 35043512 PMC8766293

[7] Frasie A Houry M Plourde C Robert MT Bouyer LJ Roy JS. Feedback for the prevention and rehabilitation of work-related musculoskeletal disorders: a systematic review. Work. (2023) 76:61–94. doi: 10.3233/wor-220545, PMID: 36872834

[8] Isusi I. Work-related musculoskeletal disorders – Facts and figures. Luxembourg: European Agency for Safety and Health at Work (2020).

[9] Engel GL. The need for a new medical model: a challenge for biomedicine. Science. (1977) 196:129–36. doi: 10.1126/science.847460, PMID: 847460

[10] Otero-Ketterer E Peñacoba-Puente C Ferreira Pinheiro-Araujo C Valera-Calero JA Ortega-Santiago R. Biopsychosocial factors for chronicity in individuals with non-specific low back pain: an umbrella review. Int J Environ Res Public Health. (2022) 19:19. doi: 10.3390/ijerph191610145, PMID: 36011780 PMC9408093

[11] Edwards RR Dworkin RH Sullivan MD Turk DC Wasan AD. The role of psychosocial processes in the development and maintenance of chronic pain. J Pain. (2016) 17:T70–92. doi: 10.1016/j.jpain.2016.01.001, PMID: 27586832 PMC5012303

[12] Rashid M Heiden M Nilsson A Kristofferzon ML. Do work ability and life satisfaction matter for return to work? Predictive ability of the work ability index and life satisfaction questionnaire among women with long-term musculoskeletal pain. BMC Public Health. (2021) 21:584. doi: 10.1186/s12889-021-10510-8, PMID: 33761910 PMC7992335

[13] Cuenca-Zaldívar JN Fernández-Carnero J Sánchez-Romero EA Álvarez-Gonzalo V Conde-Rodríguez R Rodríguez-Sanz D. Effects of a therapeutic exercise protocol for patients with chronic non-specific Back pain in primary health care: a single-group retrospective cohort study. J Clin Med. (2023) 12:12. doi: 10.3390/jcm12206478, PMID: 37892618 PMC10607108

[14] Martínez-Pozas O Sánchez-Romero EA Beltran-Alacreu H Arribas-Romano A Cuenca-Martínez F Villafañe JH. Effects of orthopedic manual therapy on pain sensitization in patients with chronic musculoskeletal pain: an umbrella review with Meta-Meta-analysis. Am J Phys Med Rehabil. (2023) 102:879–85. doi: 10.1097/phm.0000000000002239, PMID: 36917046

[15] OECD. Sickness, disability and work: Breaking the barriers: A synthesis of findings across OECD countries. Paris: OECD Publishing (2010).

[16] Fresk M Grooten WJA Brodin N Backlund LG Arrelöv B Skånér Y. Mapping information regarding the work-related disability of depression and long-term musculoskeletal pain to the international classification of functioning, disability and health and ICF Core sets. Front Rehabil Sci. (2023) 4:1159208. doi: 10.3389/fresc.2023.1159208, PMID: 37200737 PMC10185752

[17] Artiles-Sánchez J Fernández-Carnero J Sánchez-Romero EA Cuenca-Zaldívar JN Martinez-Lozano P Meléndez-Oliva E. Multicomponent exercise program to avoid productivity loss due to COVID-19: A prospective study with a brief report of 2-year follow-up. Topics Geriatr. Rehab. (2024) 40:175–83. doi: 10.1097/tgr.0000000000000439

[18] Langella F Christensen SWM Palsson TS Høgh M Gagni N Bellosta-López P. Development of the prevent for work questionnaire (P4Wq) for assessment of musculoskeletal risk in the workplace: part 1-literature review and domains selection. BMJ Open. (2021) 11:e043800. doi: 10.1136/bmjopen-2020-043800, PMID: 33846150 PMC8048000

[19] Langella F Vanni D Høgh M Palsson TS Christensen SWM Bellosta-López P. Development of the prevent for work questionnaire (P4Wq) for the assessment of musculoskeletal risk factors in the workplace: part 2-pilot study for questionnaire development and validation. BMJ Open. (2021) 11:e053988. doi: 10.1136/bmjopen-2021-053988, PMID: 34952882 PMC9066351

[20] Mokkink LB Terwee CB Patrick DL Alonso J Stratford PW Knol DL. The COSMIN checklist for assessing the methodological quality of studies on measurement properties of health status measurement instruments: an international Delphi study. Qual Life Res. (2010) 19:539–49. doi: 10.1007/s11136-010-9606-8, PMID: 20169472 PMC2852520

[21] Beaton DE Bombardier C Guillemin F Ferraz MB. Guidelines for the process of cross-cultural adaptation of self-report measures. Spine. (2000) 25:3186–91. doi: 10.1097/00007632-200012150-00014 11124735

[22] Blasco-Abadía J Bellosta-López P Palsson T Moreno González S García-Campayo J Doménech-García V. Spanish version of the pain beliefs questionnaire: translation, cross-cultural adaptation, validation, and psychometric properties in a working population. Musculoskelet Sci Pract. (2023) 66:102827. doi: 10.1016/j.msksp.2023.102827, PMID: 37459817

[23] Papaefstathiou E Tsounis A Malliarou M Sarafis P. Translation and validation of the Copenhagen burnout inventory amongst Greek doctors. Health Psychol Res. (2019) 7:7678. doi: 10.4081/hpr.2019.7678, PMID: 31583289 PMC6763708

[24] Hernandez G Garin O Pardo Y Vilagut G Pont À Suárez M. Validity of the EQ-5D-5L and reference norms for the Spanish population. Qual Life Res. (2018) 27:2337–48. doi: 10.1007/s11136-018-1877-5, PMID: 29767329

[25] Jensen MB Jensen CE Gudex C Pedersen KM Sørensen SS Ehlers LH. Danish population health measured by the EQ-5D-5L. Scand J Public Health. (2023) 51:241–9. doi: 10.1177/14034948211058060, PMID: 34847818 PMC9969307

[26] Selva-Sevilla C Ferrara P Gerónimo-Pardo M. Psychometric properties study of the Oswestry disability index in a Spanish population with previous lumbar disc surgery: homogeneity and validity. Spine. (2019) 44:E430–e437. doi: 10.1097/brs.0000000000002867 30234803

[27] Comins J Brodersen J Wedderkopp N Lassen MR Shakir H Specht K. Psychometric validation of the Danish version of the Oswestry disability index in patients with chronic low Back pain. Spine (Phila Pa 1976). (2020) 45:1143–50. doi: 10.1097/brs.0000000000003486. 32205707

[28] Coggon D Ntani G Palmer KT Felli VE Harari R Barrero LH. Disabling musculoskeletal pain in working populations: is it the job, the person, or the culture? Pain. (2013) 154:856–63. doi: 10.1016/j.pain.2013.02.008, PMID: 23688828 PMC3675684

[29] Li CH. Confirmatory factor analysis with ordinal data: comparing robust maximum likelihood and diagonally weighted least squares. Behav Res Methods. (2016) 48:936–49. doi: 10.3758/s13428-015-0619-7, PMID: 26174714

[30] Hair JF Black WC Babin BJ Anderson RE. Multivariate data analysis. 6th ed. Upper Saddle River, New Jersey: Pearson/Prentice Hall (2006).

[31] Maydeu-Olivares A Shi D Rosseel Y. Assessing fit in structural equation models: a Monte-Carlo evaluation of RMSEA versus SRMR confidence intervals and tests of close fit. Struct Equ Model Multidiscip J. (2018) 25:389–402. doi: 10.1080/10705511.2017.1389611

[32] Kenny DA Kaniskan B McCoach DB. The performance of RMSEA in models with small degrees of freedom. Sociol Methods Res. (2015) 44:486–507. doi: 10.1177/0049124114543236

[33] Bentler PM. Comparative fit indexes in structural models. Psychol Bull. (1990) 107:238–46. doi: 10.1037/0033-2909.107.2.238, PMID: 2320703

[34] Tavakol M Dennick R. Making sense of Cronbach's alpha. Int J Med Educ. (2011) 2:53–5. doi: 10.5116/ijme.4dfb.8dfd, PMID: 28029643 PMC4205511

[35] Schober P Boer C Schwarte LA. Correlation coefficients: appropriate use and interpretation. Anesth Analg. (2018) 126:1763–8. doi: 10.1213/ane.0000000000002864, PMID: 29481436

[36] Cohen J. Statistical power analysis for the behavioral sciences. New York: Routledge Academic (1988).

[37] Cohen J. A coefficient of agreement for nominal scales. Educ Psychol Meas. (1960) 20:37–46. doi: 10.1177/001316446002000104

[38] Koo TK Li MY. A guideline of selecting and reporting Intraclass correlation coefficients for reliability research. J Chiropr Med. (2016) 15:155–63. doi: 10.1016/j.jcm.2016.02.012, PMID: 27330520 PMC4913118

[39] Furlan L Sterr A. The applicability of standard error of measurement and minimal detectable change to motor learning research-a behavioral study. Front Hum Neurosci. (2018) 12:95. doi: 10.3389/fnhum.2018.00095, PMID: 29623034 PMC5875129

[40] Terwee CB Bot SD de Boer MR, van der Windt DA Knol DL Dekker J. Quality criteria were proposed for measurement properties of health status questionnaires. J Clin Epidemiol (2007) 60: 34–42. doi: 10.1016/j.jclinepi.2006.03.012, PMID: 17161752

[41] McKown S Acquadro C Anfray C Arnold B Eremenco S Giroudet C. Good practices for the translation, cultural adaptation, and linguistic validation of clinician-reported outcome, observer-reported outcome, and performance outcome measures. J Patient Rep Outcomes. (2020) 4:89. doi: 10.1186/s41687-020-00248-z, PMID: 33146755 PMC7642163

[42] Doménech-García V Skovlund SV Bellosta-López P Calatayud J López-Bueno R Andersen LL. Does the distribution of musculoskeletal pain shape the fate of long-term sick leave? A prospective cohort study with register follow-up. Pain. (2024) 165:1875–81. doi: 10.1097/j.pain.0000000000003176, PMID: 38284407 PMC11247451

[43] Sun S. Meta-analysis of Cohen’s kappa. Health Serv Outcomes Res Methodol. (2011) 11:145–63. doi: 10.1007/s10742-011-0077-3

[44] Vinstrup J Bláfoss R López-Bueno R Calatayud J Villadsen E Clausen T. Pain control beliefs predict premature withdrawal from the labor market in workers with persistent pain: prospective cohort study with 11-year register follow-up. J Pain. (2023) 24:1820–9. doi: 10.1016/j.jpain.2023.05.009, PMID: 37201673

