# Supplementary material for: Cross-cultural adaptation and validation of the Spanish version of the Prevent for Work questionnaire
Source: Front Public Health. 2025 Jan 7;12:1453492. doi: 10.3389/fpubh.2024.1453492 (PMC11747122; doi:10.3389/fpubh.2024.1453492)
Supplement: Supplementary file 1 [file Table_1.docx]

**Supplementary Table A.** Detailed description of the P4Wq and other questionnaires completed for testing construct validity.

| **Questionnaire (abbreviation)** | **Aim** | **Sections** | **Dimensions / Domains and items** | **Scoring Scale and Total Score** | **Final Score Interpretation** |
| --- | --- | --- | --- | --- | --- |
| Prevent4Work  (P4Wq) | Measure factors related to the development of work-related MSK pain | 1. Sociodemographic and disabling MSK pain prevalence in the last 12 months | Age, sex, weight, height, years in current position, and disabling MSK pain prevalence in the last 12 months [neck, shoulders, elbow, wrist/hand, dorsal region, low back, hip, knee, ankle/foot] in different body regions. | NA | NA |
|  |  | 2. Potential work-related risk factors for developing MSK pain | 1. Job satisfaction (items 1-4) 2. Mental stress (items 5-10) 3. Kinesiophobia & Catastrophizing (items 11-14) 4. Physical stress (items 15-20) | 5-point Likert Scale (0-4) with options "Never," "Rarely," "Sometimes," "Often", or "Always". | Higher scores indicate a worse scenario (i.e., potential higher risk) |
| EuroQol Five-dimensions Five-levels  (EQ-5D-5L) | Measure generic health status | 1. Health state description based on 5 dimensions | 1. Mobility 2. Self-care 3. Activity 4. Pain-Discomfort 5. Anxiety-Depression | 5-point Likert Scale (1-5) with scores of 1 indicating "absence of problems" and 5 "extreme problems". | Algorithmic calculation based on country-specific formula and methods. |
|  |  | 2. Overall health status evaluation | • General health. | 0-100 VAS with 100 indicating "best imaginable health" and 0 "worst imaginable health". | Higher scores indicate a worse scenario (i.e., potential higher risk) |
| Oswestry Disability Index  (ODI) | Assess disability related to spinal disorders | 1. Different dimensions of daily living potentially limited by the spinal disorder | • Dimensions including 10 items: pain intensity, lifting, personal care, walking, sitting, standing, sex, social life, sleeping, and travel. | 6-point Likert Scale (0-5), with the total score multiplied by 2 (0-100) | Higher scores indicate a higher level of disability (i.e., worst scenario) |
| *NA: not applicable. MSK: musculoskeletal.* | | | | | |

**Psychometric properties in subgroups**

**Internal consistency**

| **Supplementary Table B.** Internal consistency analysis for subgroups expressed in Cronbach's alpha values. | | | | | | |
| --- | --- | --- | --- | --- | --- | --- |
|  |  | Total score | JSS | MSS | KCS | PSS |
| Overall sample | | 0.91 | 0.90 | 0.85 | 0.91 | 0.88 |
| Sex | Female | 0.92 | 0.91 | 0.85 | 0.92 | 0.91 |
|  | Male | 0.89 | 0.89 | 0.83 | 0.89 | 0.81 |
| Age | <45 years | 0.91 | 0.90 | 0.85 | 0.92 | 0.88 |
|  | ≥45 years | 0.91 | 0.90 | 0.86 | 0.90 | 0.90 |
| Work type | Office | 0.88 | 0.81 | 0.80 | 0.87 | 0.91 |
|  | Healthcare | 0.93 | 0.94 | 0.88 | 0.93 | 0.88 |
|  | Blue-collar | 0.85 | 0.80 | 0.79 | 0.88 | 0.77 |
| *JSS: job satisfaction subdomain; MSS: mental stress subdomain; KCS: kinesiophobia & catastrophizing subdomain; PSS: physical stress subdomain.* | | | | | | |

**Convergent validity**

| **Supplementary Table C.** Convergent analysis for subgroups for the P4Wq total score expressed in Pearson’s coefficients. | | | | |
| --- | --- | --- | --- | --- |
|  |  | EQ-VAS | ODI |  |
| Overall sample | | -0.43 | 0.46 |  |
| Sex | Female | -0.41 | 0.55 |  |
|  | Male | -0.56 | 0.43 |  |
| Age | <45 years | -0.44 | 0.40 |  |
|  | ≥45 years | -0.43 | 0.49 |  |
| Work type | Office | -0.36 | 0.44 |  |
|  | Healthcare | -0.45 | 0.36 |  |
|  | Blue-collar | -0.48 | 0.47 |  |
| *P4Wq: Prevent4Work questionnaire; EQ-VAS: EuroQol visual analogue scale for self-percieved general health status; ODI: Oswestry Disability Index.* | | | | |

**Following COSMIN recommendations, other psychometric properties (i.e., structural validity or test-retest reliability) were not assessed in the subgroups due to the reduced sample size when subgrouping.**

**Subgroups analysis: sex (male or female)**

| **Supplementary Table D.** P4Wq scores comparisons between male and female. | | | |  |
| --- | --- | --- | --- | --- |
|  | Female (n=97) | Male (n=57) | p value | |
| Total score (0/80) | 29.3 ± 14.4 | 32.6 ± 13.6 | 0.165 | |
| JSS (0/16) | 5.8 ± 4.1 | 6.2 ± 4.2 | 0.571 | |
| MSS (0/24) | 9.2 ± 4.7 | 8.9 ± 5.0 | 0.695 | |
| KCS (0/16) | 5.7 ± 4.4 | 6.2 ± 4.3 | 0.519 | |
| PSS (0/24) | 8.5 ± 5.9 | 11.3 ± 5.1 | 0.004 | |
| *Data are expressed in median ± standard deviation. P4Wq: Prevent4Work questionnaire; JSS: job satisfaction subdomain; MSS: mental stress subdomain; KCS: kinesiophobia & catastrophizing subdomain; PSS: physical stress subdomain. P values represent significance level after independent samples T-test.* | | | |  |

**Subgroups analysis: age categories (<45 years or ≥45 years)**

| **Supplementary Table E.** P4Wq scores comparisons between age categories. | | | |  |
| --- | --- | --- | --- | --- |
|  | <45 years (n=97) | ≥45 years (n=53) | p value | |
| Total score (0/80) | 30.7 ± 14.7 | 30.5 ± 13.6 | 0.916 | |
| JSS (0/16) | 5.9 ± 4.2 | 6.2 ± 4.1 | 0.687 | |
| MSS (0/24) | 9.2 ± 5.1 | 9.0 ± 4.4 | 0.797 | |
| KCS (0/16) | 5.6 ± 4.6 | 6.5 ± 4.2 | 0.197 | |
| PSS (0/24) | 10.0 ± 5.8 | 8.7 ± 5.6 | 0.181 | |
| *Data are expressed in median ± standard deviation. P4Wq: Prevent4Work questionnaire; JSS: job satisfaction subdomain; MSS: mental stress subdomain; KCS: kinesiophobia & catastrophizing subdomain; PSS: physical stress subdomain. P values represent significance level after independent samples T-test.* | | | |  |

**Subgroups analysis: work type (office workers, healthcare workers, blue-collar workers)**

| **Supplementary Table F.** P4Wq scores comparisons between work types. | | | | |  |
| --- | --- | --- | --- | --- | --- |
|  | Office workers (n=42) | Healthcare workers (n=56) | Blue-collar workers (n=55) | p value | |
| Total score (0/80) | 21.6 ± 9.4 | 36.1 ± 16.3* | 31.8 ± 11.4* | <0.001 | |
| JSS (0/16) | 4.3 ± 2.5*^#^* | 8.0 ± 5.1 | 5.3 ± 3.2*^#^* | <0.001 | |
| MSS (0/24) | 8.3 ± 3.5*^#^* | 11.0 ± 5.2 | 7.7 ± 4.6*^#^* | <0.001 | |
| KCS (0/16) | 4.4 ± 3.5*^§^* | 5.9 ± 5.0 | 7.0 ± 4.1 | 0.013 | |
| PSS (0/24) | 4.5 ± 4.2 | 11.2 ± 5.3* | 11.8 ± 4.8* | <0.001 | |
| *Data are expressed in median ± standard deviation. P4Wq: Prevent4Work questionnaire; JSS: job satisfaction subdomain; MSS: mental stress subdomain; KCS: kinesiophobia & catastrophizing subdomain; PSS: physical stress subdomain. P values represent significance level after ANOVA test.*  ** Significant differences compared to office workers after Bonferroni correction.*  *^#^ Significant differences compared to healthcare workers after Bonferroni correction.*  *^§^ Significant differences compared to blue-collar workers after Bonferroni correction.* | | | | |  |
