# Supplementary material for: Cross-cultural adaptation and validation of the Spanish version of the Prevent for Work questionnaire
Source: Front Public Health. 2025 Jan 7;12:1453492. doi: 10.3389/fpubh.2024.1453492 (PMC11747122; doi:10.3389/fpubh.2024.1453492)
Supplement: Supplementary file 3 [file Data_Sheet_2.docx]

**Prevent4Work questionnaire (P4Wq) – Spanish version**

El siguiente cuestionario ha sido diseñado para evaluar los factores de riesgo relacionados con el trabajo que intervienen en los trastornos musculoesqueléticos, incluyendo factores de naturaleza física y psicosocial.

| Sexo: | [ ] Hombre [ ] Mujer |
| --- | --- |
| Año de nacimiento: | __________ |
| ¿Cuánto llevas desempeñando tu actual tipo de trabajo? | ____ años ______ meses |
| ¿Cuánto pesas? | __________ Kg |
| ¿Cuánto mides? | __________ cm |
| En los últimos 12 meses, ¿has tenido alguna vez problemas (incomodidad, dolor o malestar) en las siguientes regiones del cuerpo, que hayan limitado tus actividades diarias? |  |
| - Cuello | [ ] No [ ] Sí |
| - Uno o ambos hombros | [ ] No [ ] Sí |
| - Uno o ambos codos | [ ] No [ ] Sí |
| - Una o ambas muñecas/manos | [ ] No [ ] Sí |
| - Región dorsal | [ ] No [ ] Sí |
| - Región lumbar | [ ] No [ ] Sí |
| - Una o ambas caderas/muslos | [ ] No [ ] Sí |
| - Una o ambas rodillas | [ ] No [ ] Sí |
| - Uno o ambos tobillos/pies | [ ] No [ ] Sí |

Por favor, lee atentamente cada pregunta y responde seleccionando la opción que mejor refleje **tu experiencia en el lugar de trabajo.** Elije solamente una opción por pregunta, teniendo en cuenta tu puesto de trabajo actual. Es importante que respondas todas las preguntas, **incluso si nunca has tenido dolencias**.

|  |  | **Nunca** | **Rara vez** | **Alguna vez** | **A menudo** | **Siempre** |
| --- | --- | --- | --- | --- | --- | --- |
| 1 | ¿Tu trabajo te da la oportunidad de mejorar tus habilidades? | 4 | 3 | 2 | 1 | 0 |
| 2 | ¿Te sientes motivado e involucrado en tu trabajo? | 4 | 3 | 2 | 1 | 0 |
| 3 | ¿Existe una buena cooperación entre tus compañeros de trabajo? | 4 | 3 | 2 | 1 | 0 |
| 4 | ¿Estás contento con las personas con las que trabajas? | 4 | 3 | 2 | 1 | 0 |
| 5 | En las últimas 4 semanas, ¿te has sentido calmado y tranquilo? | 4 | 3 | 2 | 1 | 0 |
| 6 | Tengo dificultades para relajarme o divertirme. | 0 | 1 | 2 | 3 | 4 |
| 7 | No soy capaz de plantar cara a los demás. | 0 | 1 | 2 | 3 | 4 |
| 8 | Tengo dificultades para ser feliz. | 0 | 1 | 2 | 3 | 4 |
| 9 | En las últimas 4 semanas, ¿has tenido problemas de concentración? | 0 | 1 | 2 | 3 | 4 |
| 10 | En las últimas 4 semanas, ¿has tenido dificultad para pensar con claridad? | 0 | 1 | 2 | 3 | 4 |
| 11 | Evito hacer movimientos innecesarios para prevenir que el dolor aumente. | 0 | 1 | 2 | 3 | 4 |
| 12 | Tengo miedo de que el dolor pueda aumentar. | 0 | 1 | 2 | 3 | 4 |
| 13 | Tengo la sensación de no poder soportar más el dolor. | 0 | 1 | 2 | 3 | 4 |
| 14 | El dolor es terrible, y pienso que no mejorará nunca. | 0 | 1 | 2 | 3 | 4 |
| 15 | ¿En tu trabajo debes levantar cargas pesadas (más de 5 kg)? | 0 | 1 | 2 | 3 | 4 |
| 16 | ¿En tu trabajo debes levantar cargas desde una postura incómoda? | 0 | 1 | 2 | 3 | 4 |
| 17 | ¿En tu trabajo debes doblar y girar (al mismo tiempo) el tronco? | 0 | 1 | 2 | 3 | 4 |
| 18 | ¿En tu trabajo debes levantar los brazos por encima de la altura de los hombros? | 0 | 1 | 2 | 3 | 4 |
| 19 | ¿En tu trabajo debes mantener posturas incómodas? | 0 | 1 | 2 | 3 | 4 |
| 20 | ¿En tu trabajo tienes dificultad para ejercer suficiente fuerza debido a posturas incómodas? | 0 | 1 | 2 | 3 | 4 |
